# PED in 2024: improving the community deposition of structural ensembles for intrinsically disordered proteins

**DOI:** 10.1093/nar/gkad947

**Published:** 2023-10-30

**Authors:** Hamidreza Ghafouri, Tamas Lazar, Alessio Del Conte, Luiggi G Tenorio Ku, PED Consortium, Maria C Aspromonte, Pau Bernadó, Belén Chaves-Arquero, Lucia Beatriz Chemes, Damiano Clementel, Tiago N Cordeiro, Carlos A Elena-Real, Michael Feig, Isabella C Felli, Carlo Ferrari, Julie D Forman-Kay, Tiago Gomes, Frank Gondelaud, Claudiu C Gradinaru, Tâp Ha-Duong, Teresa Head-Gordon, Pétur O Heidarsson, Giacomo Janson, Gunnar Jeschke, Emanuela Leonardi, Zi Hao Liu, Sonia Longhi, Xamuel L Lund, Maria J Macias, Pau Martin-Malpartida, Davide Mercadante, Assia Mouhand, Gabor Nagy, María Victoria Nugnes, José Manuel Pérez-Cañadillas, Giulia Pesce, Roberta Pierattelli, Damiano Piovesan, Federica Quaglia, Sylvie Ricard-Blum, Paul Robustelli, Amin Sagar, Edoardo Salladini, Lucile Sénicourt, Nathalie Sibille, João M C Teixeira, Thomas E Tsangaris, Mihaly Varadi, Peter Tompa, Silvio C E Tosatto, Alexander Miguel Monzon

**Affiliations:** Department of Biomedical Sciences, University of Padova, Padova, Italy; VIB-VUB Center for Structural Biology, Vlaams Instituut voor Biotechnologie (VIB), Brussels, Belgium; Structural Biology Brussels, Department of Bioengineering, Vrije Universiteit Brussel (VUB), Brussels, Belgium; Department of Biomedical Sciences, University of Padova, Padova, Italy; Department of Biomedical Sciences, University of Padova, Padova, Italy; VIB-VUB Center for Structural Biology, Vlaams Instituut voor Biotechnologie (VIB), Brussels, Belgium; Structural Biology Brussels, Department of Bioengineering, Vrije Universiteit Brussel (VUB), Brussels, Belgium; Institute of Enzymology, Research Centre for Natural Sciences (RCNS), Budapest, Hungary; Department of Biomedical Sciences, University of Padova, Padova, Italy; Department of Information Engineering, University of Padova, Padova, Italy

## Abstract

The Protein Ensemble Database (PED) (URL: https://proteinensemble.org) is the primary resource for depositing structural ensembles of intrinsically disordered proteins. This updated version of PED reflects advancements in the field, denoting a continual expansion with a total of 461 entries and 538 ensembles, including those generated without explicit experimental data through novel machine learning (ML) techniques. With this significant increment in the number of ensembles, a few yet-unprecedented new entries entered the database, including those also determined or refined by electron paramagnetic resonance or circular dichroism data. In addition, PED was enriched with several new features, including a novel deposition service, improved user interface, new database cross-referencing options and integration with the 3D-Beacons network—all representing efforts to improve the FAIRness of the database. Foreseeably, PED will keep growing in size and expanding with new types of ensembles generated by accurate and fast ML-based generative models and coarse-grained simulations. Therefore, among future efforts, priority will be given to further develop the database to be compatible with ensembles modeled at a coarse-grained level.

## Graphical Abstract

**Graphical Abstract:**
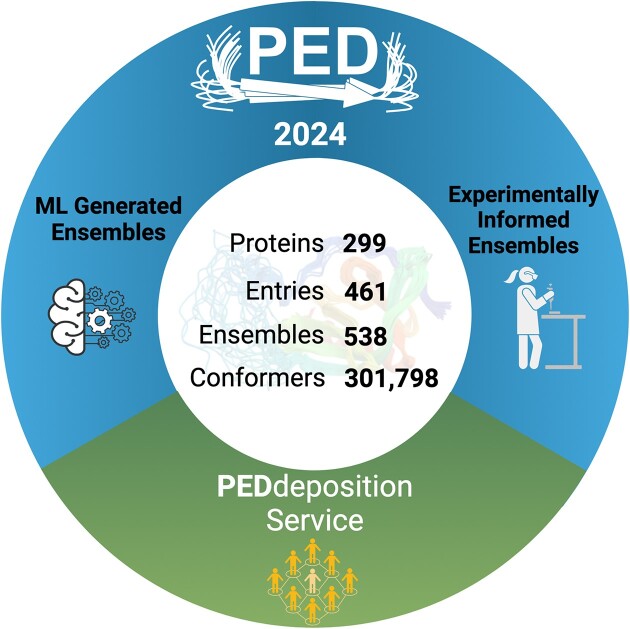

## Introduction

Intrinsically disordered proteins or regions (IDPs/IDRs) lack a specific, stable structure and instead exist as rapidly interconverting conformers. This arises from their relatively uniform free-energy landscape, resulting in their highly dynamic and heterogeneous nature (1). IDPs/IDRs play significant roles in various essential functions such as cell signaling, regulation and recognition. Furthermore, their involvement in numerous human diseases renders them highly attractive targets for therapeutic drug discovery (2). While binding modes of some IDPs/IDRs that fold upon interaction offer valuable structural insights (3), gaining a thorough comprehension of the complex mechanisms governing the function of IDPs also requires knowledge of their structural dynamics in the unbound state, and many IDPs/IDRs form fuzzy complexes (3). Given their extreme conformational dynamics, modeling IDPs/IDRs in terms of ensembles is the only valid strategy for structurally studying IDP function. By definition, a conformational ensemble consists of multiple structures, each with their statistical weights representing their relative populations and transition rates that quantify their dynamics (4). Despite the steady expansion of experimentally determined protein structures in the Protein Data Bank (5) and the recent AlphaFold Protein Structure Database (6), which contains accurate structural models of millions of proteins, the information they offer about the dynamic nature of proteins remains limited, especially in the context of ensemble representation of IDPs. In 2014, the Protein Ensemble Database (PED) (7) was established to bridge this gap, and over time, it has consistently evolved, enhancing the quantity and quality of deposited ensembles.

Generally, conformational ensembles are determined by integrating experimental and computational methods. This involves a diverse range of experimental techniques, including nuclear magnetic resonance (NMR) spectroscopy, small angle X-ray scattering (SAXS), single-molecule Förster resonance energy transfer (smFRET), electron paramagnetic resonance (EPR) and circular dichroism (CD) (4,8). These experimental measurements serve as global and/or local constraints, enabling the resampling and reweighting of a pool of conformers generated through statistical conformer generators or molecular dynamics (MD)/Monte Carlo (MC) simulations. Moreover, the advent of AlphaFold2 (9), RoseTTAFold (10) and the advancements in machine learning approaches have fostered the development of various pipelines aimed at effectively modeling multiple conformational states or predicting conformational ensembles (11–13). Nevertheless, despite recent progress in the field, modeling conformational ensembles, especially for IDRs/IDPs, remains challenging. On the computational front, a significant obstacle arises from the lack of a precise energy function to guide MD or MC simulations (14,15), coupled with limited computational resources for thorough sampling of the conformational space (16). On the other hand, from an experimental standpoint, a major challenge is accurately quantifying all sources of errors and uncertainties in both the experimental data and the predictors (forward models). Additionally, the observable data are averaged over all members of the ensemble, leading to a reduction in information content. Because of these limitations, resolving structural ensembles has persisted as an ‘underdetermined’ challenge. This viewpoint arises from the fact that the number of degrees of freedom in the ensembles significantly surpasses the available experimental restraints, leading to multiple potential solutions for the problem without a distinct ‘best’ option. In such a context, having comprehensive and manually curated IDP-related databases, e.g. PED (17), DisProt (18), MobiDB (19), FuzDB (3) and IDEAL (20), can serve multiple purposes. First and foremost, they can serve as a foundational reference and a valuable resource for establishing a validation pipeline to assess the reliability of IDP conformational ensembles. Furthermore, they function as extensive training datasets for upcoming machine learning (ML) models (21). Since the latest PED publication in 2021 (17), a strong emphasis has been given by the scientific community to predict IDP conformational ensembles from sequence by combining ML approaches and MD simulations (22,23), as well as to compare conformational ensembles of flexible proteins (24,25). In this article, we present the new version of the PED (Protein Ensemble Database, https://proteinensemble.org), aimed at addressing the evolving challenges and advancements in the field of IDPs/IDRs. Our primary goal has consistently been to enhance the size of our database. In this updated release of PED, we have now accumulated a total of 461 entries and 538 ensembles. This time, we also included IDP ensembles generated without experimental data by novel ML and sampling methods from sequences. A new restyled website with an improved user interface and novel features is presented, as well as a dedicated web-server for the ensemble's deposition and curation.

### Progress and new features

#### Database growth

PED aims to be the gold-standard primary deposition database for conformational ensembles of non-globular proteins (NGPs) or regions. Therefore, the main goal of PED is to provide an ever-growing platform of structural ensemble entries with a user-friendly deposition pipeline while maintaining high standards for data quality and the FAIR data principles. PED is cross-linked with the main resources to deposit ensembles’ primary experimental data, including BRMB (26), SASBDB (27) and PCDDB (28).

In this new release, the number of PED entries has increased almost three times compared to the version presented in the last publication (461 versus 162) (17). The source of this data increment comes from depositions from data owners (42 entries in this release), ensembles generated without experimental data (61 entries in this release) and ensemble identification by the PED biocurator team from databases and publications. As detailed below, a larger number of NMR ensemble entries were identified by an automated computational pipeline applied to BMRB (1409 protein structures), which were then subsequently revised, filtered and published by the biocurators (totalling 189 entries).

#### New entries

##### Novel ensembles

**Figure 1.**
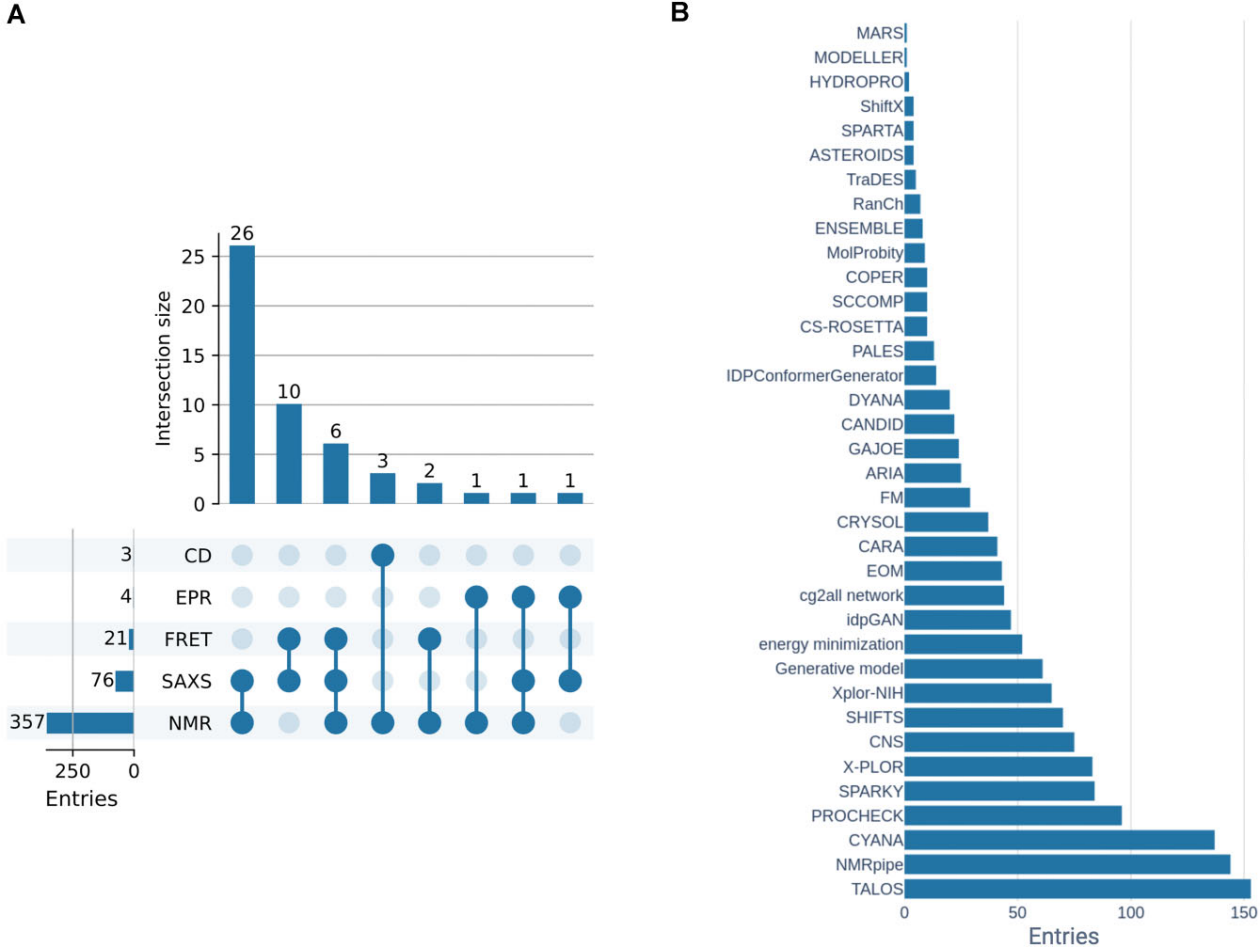
Experimental techniques and ensemble generation methods. (**A**) Matrix layout quantifies the combinations of experimental techniques for PED entries, sorted by size. Filled circles in the matrix indicate which experimental measurement is part of the intersection. (**B**) Distribution of ensemble generation methods and auxiliary software applied in PED. The X axis represents the number of PED entries.

As in previous releases, new PED ensembles were predominantly modeled using SAXS, NMR spectroscopy, FRET data and their combinations (Figure 1A). NMR data included chemical shifts (CSs), nuclear Overhauser effects (NOEs), J-couplings, residual dipolar couplings (RDCs), relaxation data and paramagnetic relaxation enhancements (PREs) (29). On top of these, for a few new entries, methods such as electron paramagnetic resonance (EPR) spectroscopy techniques (e.g. double electron–electron resonance (DEER)) and CD were also used to characterize the protein ensembles (30,31); often in combination with other techniques. These combinations included EPR + NMR, EPR + SAXS, EPR + NMR + SAXS, CD + NMR (30–32). NMR data have already been cross-referenced from BMRB (26) and SAXS data from SASBDB (27), but now CD data can also be cross-referenced from PCDDB (28), which will enable PED depositors during submission to reference their CD data already deposited in its primary resource.

Besides these experimental datasets, several PED depositions also used computationally expensive MD simulations to perform integrative structural modeling by reweighting the ensembles. These MD simulations comprised among others trajectories generated by a CHARMM force field and the EEF1 implicit solvent model in a replica-exchange MD setup (33), or AMBER03w force field with TIP4P/2005s water model (34), or replica-exchange Discrete MD (DMD) using the MEDUSA force field in implicit water (35), or coarse-grained Langevin MD in multiple replicas (36), or AMBER99SB-disp force field with it own water model using replica exchange with solute tempering (37). Furthermore, the repertoire of ensemble generation methods and auxiliary software is continuously expanding to encompass state-of-the-art techniques in the field (Figure 1B).

It is often emphasized that IDPs have a high-degree of conformational heterogeneity, which is harder to capture by a single technique. Therefore the integration of simulations and various experiments can better characterize the highly dynamic nature of IDPs (38). Now, there is an increasing number of IDP ensembles in PED determined by different combinations of techniques under the same or slightly different conditions, e.g. hnRNPA1, alpha-synuclein, Tau. We envisage that these ensemble data will reveal not only how sensitive IDPs are to environmental conditions but also the strengths and weaknesses of methods in capturing certain structural aspects.

##### NMR structural ensembles

A significant upgrade in the new PED version involves the inclusion of a large number of NMR structural ensembles comprising IDRs sourced from the PDB. These NMR ensembles represent collections of different conformations (models) that individually satisfy the experimentally derived constraints (8).

To achieve this, we systematically searched the MobiDB (19) to identify NMR ensembles containing IDRs/IDPs. As a starting point, we identified a subset of 2064 proteins containing large RMSD regions defined as ‘mobile’ in MobiDB. Mobile regions are calculated for all NMR ensembles using the Mobi software (39); it is an analogous definition to the presence of missing residues in X-ray structures. This feature represents highly flexible regions based on structural superposition that change their local conformation in the NMR ensembles.

To further refine our dataset, we also consider the disorder content percentage of the proteins based on two criteria: AlphaFold-disorder (40) and MobiDB-lite predictions (41). Initially, we focused on proteins for which both predictors indicated a disorder content percentage exceeding 50%. In the subsequent phase, we expanded our inclusion criteria to cover proteins where at least one of these two predictors indicated a disorder content percentage above 50%. During this stage, we also verified the availability of experimental NMR data for each protein in the BMRB database (26).

We then established three key criteria to determine the eligibility of NMR ensembles for inclusion in PED: (i) publication availability: we confirmed the existence of a corresponding publication; (ii) consistency in disorder prediction: we ensured that a minimum of ten consecutive residues within the mobile region were classified as disordered by AlphaFold-disorder and/or MobiDB-lite, the cutoff representing the minimum length of IDRs in DisProt; (iii) sufficient conformational coverage: the NMR ensemble had to consist of at least ten distinct structures.

#### Ensembles without explicit experimental data

Given the recent advancements in ML algorithms for modeling protein structural dynamics (42) and in new methods for sampling IDP conformational ensembles (43,44), we expanded PED and its controlled vocabulary (CV) (https://proteinensemble.org/about) to accommodate ensembles calculated without incorporating specific experimental data constraints.

The idpGAN generative model (22) was trained on coarse-grained molecular dynamics (MD) simulations (45) of IDRs from the DisProt database. It is capable of rapidly generating ensembles for arbitrary IDR sequences. IdpGAN does not incorporate experimental data in the ensemble-generation process and, for this update, we did not adopt any reweighting scheme (4) to improve compatibility with the experimental data of the entries. idpGAN was applied to a specific set of sequences from the PED database, involving the careful selection of 47 entries meeting both idpGAN’s technical prerequisites and exhibiting a significant fraction of disorder. In this context, idpGAN generated 1000 Cα-only conformers for each selected entry, which were then converted into full all-atom structures using the cg2all neural network (46). These resulting structures underwent an energy minimization relaxation process similar to the one in AF predictions. For reproducibility, the entire pipeline was made accessible at https://github.com/feiglab/idpgan_ped.

We have also included fourteen ensembles generated with the new IDPConformerGenerator software suite, which allows statistical or experimentally (chemical shift) biased sampling of torsion angles from the PDB to create all-atom IDPs and IDRs (tails, linkers and loops) in the context of full-length proteins containing folded domains (43,44). IDPConformerGenerator allows exploration of multiple torsion-angle sampling methods that enrich the ensembles’ conformational diversity and account for post-translational modifications, multi-chain protein complexes, non-protein ligands such as nucleic acids and lipid bilayers around membrane-bound proteins containing IDRs. The ensembles deposited were assessed in the original publications (43,44). IDPConformerGenerator is open-source, fully documented with examples, and is accessible at https://github.com/julie-forman-kay-lab/IDPConformerGenerator.

#### PEDdeposition service

**Figure 2.**
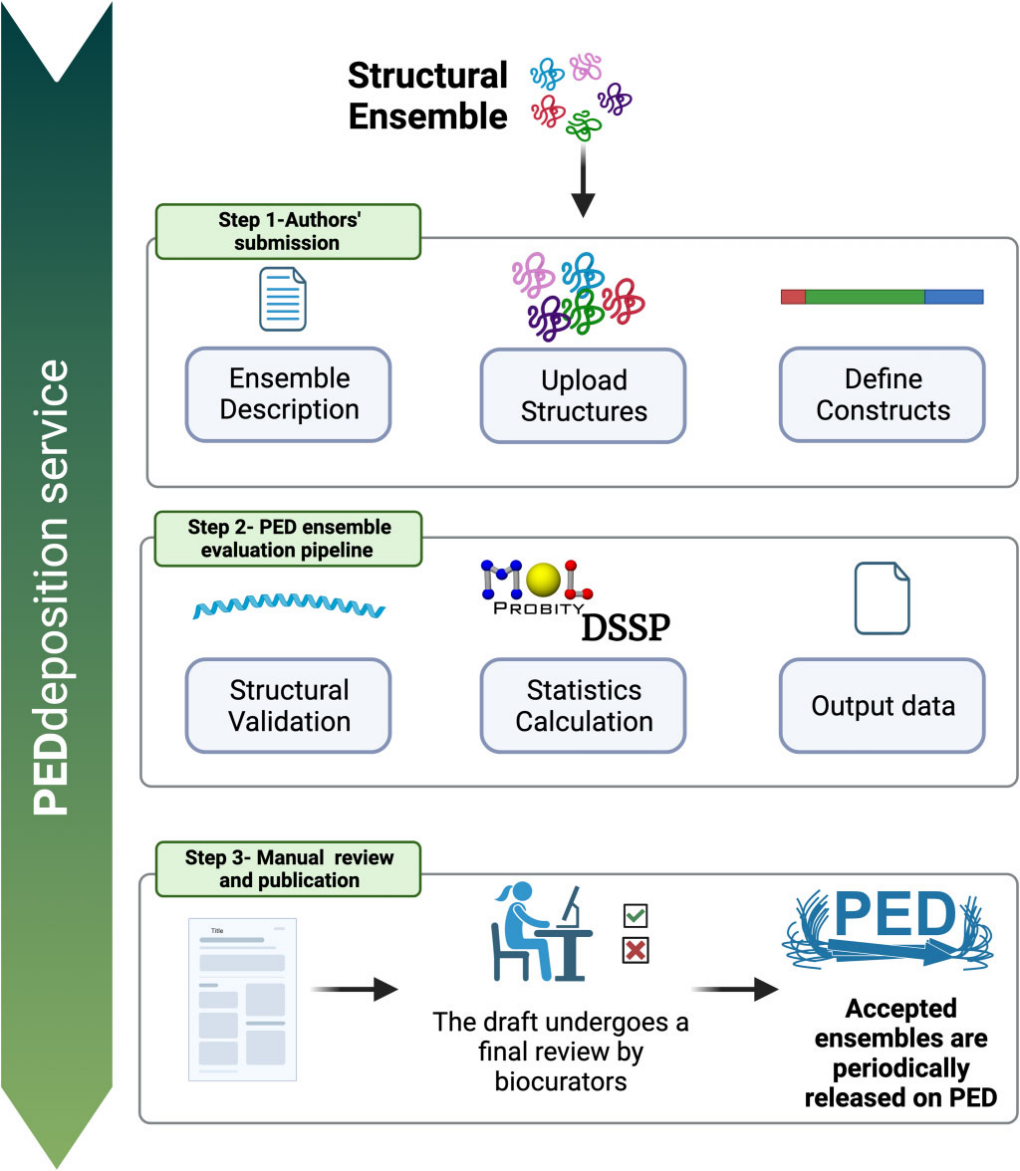
PEDdeposition service workflow. The workflow begins with the submission of an ensemble, which includes the description of both experimental and computational components, the deposition of conformers and the specification of the protein construct via UniProt accessions and/or protein sequence. The next step involves running the validation pipeline to evaluate the uploaded structures and generating insightful statistics through tools such as MolProbity, DSSP and calculating the radius of gyration. At the final stage, the submitted ensemble entry undergoes a final review by biocurators who determine whether to accept or reject it. Ultimately, approved ensembles are subsequently published on PED for public access.

PED introduces a dedicated deposition user interface accessible to everyone. This service allows depositors to upload ensembles and metadata, calculate and visualize structural features, and assess ensemble quality through automated validation. Deposition of new ensembles into the PED can be described in three main stages: ensemble deposition by the user, calculation of ensemble properties, and finally, manual curation by PED expert curators (Figure 2).

The initial step in submitting an ensemble involves user authentication through ORCID ID credentials. Within the deposition service, users encounter two primary sections: one for creating a new ensemble draft and another for managing existing drafts. Additionally, the service offers an example ensemble draft to help users become acquainted with the required deposition information. After creating a new draft, the user can begin depositing information, which is organized into three main tabs: description, ensemble upload and construct definition.

##### Ensemble description

In the ‘Experimental procedure’ section, users can provide a brief overview of the experimental techniques employed to determine the protein's structural characteristics. The ‘Structural ensemble calculation’ field captures computational methods, including software for pool generation, forward models and tools for fitting experimental observables with back-calculated measurements from predicted models, as well as validation efforts on the ensemble. For ensembles generated through MD/MC simulations, there is a specific section to detail simulation parameters like software, force field and water model, simulation duration, enhanced sampling, clustering of frames, etc. Additionally, the database offers a controlled vocabulary (CV) organized into an ontology to enhance searchability and standardize keywords describing experimental methodologies, ensemble generation and MD/MC simulations. The last two sections in the ensemble description focus on specifying the NCBI taxonomy ID of the expression organism and providing cross-references to other databases, including the BMRB, SASBDB, PCDDB, DisProt and IntAct (47).

### Upload

The upload section of the PEDdeposition service facilitates efficient submission of ensembles. Users can upload multiple-model PDB files that contain the ensemble. Additionally, if available, they can upload a tab-separated file containing weights. These weights indicate the percentage contribution of each conformer to the ensemble. The PED deposition service initiates a validation pipeline to ensure accurate data formatting. External tools like DSSP (48) and MolProbity (49) are employed to compute essential parameters, including secondary structure propensity, accessible surface area, radius of gyration, Ramachandran outliers and steric clash analysis. All resultant data are made available for download.

### Construct

Here, users define constructs corresponding to the deposited protein or region. Constructs are assembled from ‘fragments’ which can be defined using UniProt accession numbers, isoform identifiers and regions. For engineered constructs, manual input of the sequence is also an option. The feature viewer highlights deviations and modifications in the sequence, aiding in accurate definition.

### Manual curation and validation

The final stage involves expert review and validation. The PED deposition service distinguishes between general depositors and expert biocurators. Biocurators have access to a dashboard where all deposited ensembles are organized based on their review status. Upon submission, deposited information undergoes thorough review and validation. If accepted, the ensemble draft is prepared for release in the PED database; if not, depositors are promptly informed of the reasons for rejection. This automated process significantly reduces the time between ensemble deposition and publication, streamlining the entire workflow.

## Implementation

The newly re-designed user interface (UI) provides a more enriched user experience and notable features. A prominent new addition to this UI version is a feature allowing users to access supplementary data from the ensemble's deposition phase. This represents a departure from the traditional report in PDF format, as users can now leverage a multitude of data assets available in CSV or JSON formats. This transition empowers users to have more flexibility to access and work with the ensemble data according to their particular needs. Furthermore, these data assets are also available on an improved REST API for programmatic access. Constructed using the Django REST framework in Python, this REST API is meticulously documented according to the OpenAPI 3.0 standard. This documentation is presented using Swagger UI, enabling users to interact with the API on the website (https://proteinensemble.org/api). The API allows users to selectively download specific data of interest, providing programmatic access to all PED data. PED also introduces a minor redesign of the browse page by grouping proteins for each entry, mitigating redundancy during the exploration of the database content. Another feature in this new version is the integration of PED in the 3D-Beacons (50), a network that provides programmatic access to macromolecular data from different data resources, therefore PED data is now also available through the 3D-Beacons API.

PED introduces a dedicated deposition interface that is accessible to all users. This new service enables depositors to measure the quality of their ensembles through an automated validation process utilizing calculations provided by tools such as MolProbity and DSSP. These calculations are facilitated by our distributed system, efficiently managing the computational workload through SLURM, enabling parallel calculation of multiple ensembles. The results are delivered in CSV and JSON formats, similar to the main user interface mentioned earlier. The service is integrated with the ORCID authentication service, utilizing the OpenID standard to verify user identity. This authentication allows users to track the status of their uploaded ensembles, which will undergo manual validation by curators before being published in the main database for public access. During this process, depositors must provide an accurate description of their ensembles and cross-reference them with other databases to enhance findability.

## Conclusions and future work

Over the past 3 years, research on IDPs/IDRs has made significant progress, marked by the introduction of a diverse range of novel computational, experimental and ML-derived techniques for resolving structural ensembles. In alignment with the most recent advancements in this field, we remain devoted to customizing the database to the community's needs. Through a large community effort, the PED has experienced a substantial increase in its repertoire, with a noteworthy rise in the number of ensembles, entries and conformers. The repertoire has also expanded with an ever-growing range of different methods and their diverse combinations, recently enriched in EPR spectroscopy. Furthermore, we have integrated NMR-derived ensembles of IDPs/IDRs into PED and generated structural ensembles using advanced ML and sampling techniques without biasing them with experimental data.

Another key improvement in this release is the complete re-implementation and redesign of the PED deposition service. This tool has evolved beyond its previous capabilities, and now offers depositors a user-friendly, step-by-step workflow for retaining their structural ensembles. Furthermore, it includes a fully automated validation pipeline that comprehensively assesses the structure file format and generates insightful statistics. Additionally, the validation pipeline is now accessible as a standalone resource, enabling anyone interested to assess the quality of structural ensembles independently.

Further developments must be made in the future to address several key areas. To begin with, there is a need to smoothly integrate coarse-grained (CG) ensembles into PED. Recently, a pair of IDP force fields have emerged that efficiently generate CG models of random coil-like IDPs/IDRs and capture the global characteristics of disordered proteins, such as the radius of gyration (23,51–53). The integration of such models into PED is long-awaited and has the potential to significantly expand the number of available ensembles for IDPs/IDRs.

The need for a conceptual categorization of entries within PED becomes increasingly important as we progress towards incorporating *ab-initio* and NMR ensembles, and in the near future, CG ensembles. Furthermore, by including predicted ensembles by diverse algorithms, we can facilitate the benchmarking and comparison of various ensemble generation methods for IDPs. Recently, the rapid growth of DisProt enabled the design of two community benchmark efforts, termed Critical Assessment of Protein Intrinsic Disorder prediction (CAID) challenge (website: https://caid.idpcentral.org/challenge) (54–56). We envision that the consistent growth of PED will also facilitate organizing a similar benchmark using withheld high-quality ensemble data and promote the development of predictors for IDP structural ensembles.

The long-term sustainability of PED is ensured by its central role in various initiatives involving large communities of bioinformaticians and structural biologists working in the disordered proteins field. Such communities include the ‘ML4NGP’ COST Action and the ELIXIR IDP Community, both of which foster collaboration and knowledge exchange among experts.

## Data Availability

The data that support the findings of this study are openly available in PED at https://proteinensemble.org/.

## Appendix

### PED Consortium

Maria C. Aspromonte^1^, Pau Bernadó^6^, Belén Chaves-Arquero^7^, Lucia Beatriz Chemes^8^, Damiano Clementel^1^, Tiago N. Cordeiro^9^, Carlos A. Elena-Real^6^, Michael Feig^10^, Isabella C. Felli^11^, Carlo Ferrari^5^, Julie D. Forman-Kay^12,13^, Tiago Gomes^9,26^, Frank Gondelaud^14^, Claudiu C. Gradinaru^15,16^, Tâp Ha-Duong^17^, Teresa Head-Gordon^18,19,20,21^, Pétur O. Heidarsson^22^, Giacomo Janson^10^, Gunnar Jeschke^23^, Emanuela Leonardi^1^, Zi Hao Liu^12,13^, Sonia Longhi^14^, Xamuel L. Lund^6,24^, Maria J Macias^25,26^, Pau Martin-Malpartida^26^, Davide Mercadante^27^, Assia Mouhand^6^, Gabor Nagy^28^, María Victoria Nugnes^1^, José Manuel Pérez-Cañadillas^29^, Giulia Pesce^14^, Roberta Pierattelli^11^, Damiano Piovesan^1^, Federica Quaglia^1,30^, Sylvie Ricard-Blum^31^, Paul Robustelli^32^, Amin Sagar^6^, Edoardo Salladini^33^, Lucile Sénicourt^6^, Nathalie Sibille^6^, João M. C. Teixeira^12^, Thomas E. Tsangaris^15,16^, Mihaly Varadi^34^

^6^ Centre de Biologie Structurale (CBS), Université de Montpellier, INSERM, CNRS

^7^ Centro de Investigaciones Biológicas Margarita Salas (CIB), CSIC, 28040 Madrid, Spain

^8^ Instituto de Investigaciones Biotecnológicas, Universidad Nacional de San Martín (UNSAM) – Consejo Nacional de Investigaciones Científicas y Técnicas (CONICET), San Martín, Argentina

^9^ Instituto de Tecnologia Química e Biológica António Xavier, Universidade Nova de Lisboa, Oeiras, Portugal

^10^ Department of Biochemistry and Molecular Biology, Michigan State University, USA

^11^ Department of Chemistry ‘Ugo Schiff’ and Magnetic Resonance Center (CERM), University of Florence, Florence, Italy

^12^ Molecular Medicine Program, The Hospital for Sick Children, Toronto, Ontario Canada

^13^ Department of Biochemistry, University of Toronto, Toronto, Ontario, Canada

^14^ Lab. Architecture et Fonction des Macromolécules Biologiques (AFMB), UMR 7257, Aix Marseille University and Centre National de la Recherche Scientifique (CNRS), 163 Avenue de Luminy, Case 932, 13288, Marseille, FRANCE

^15^ Department of Physics, University of Toronto, Toronto, Ontario M5S 1A7, Canada

^16^ Department of Chemical & Physical Sciences, University of Toronto Mississauga, Mississauga, Ontario L5L 1C6, Canada

^17^ BioCIS, CNRS, Université Paris-Saclay, France

^18^ Kenneth S. Pitzer Center for Theoretical Chemistry, University of California, Berkeley, California, USA

^19^ Department of Chemistry, University of California, Berkeley, California, USA

^20^ Department of Chemical and Biomolecular Engineering, University of California, Berkeley, California, USA

^21^ Department of Bioengineering, University of California, Berkeley, California, USA

^22^ Department of Biochemistry, Science Institute, University of Iceland, Reykjavík, Iceland

^23^ Department of Chemistry and Applied Biosciences, ETH Zürich, Zürich, Switzerland

^24^ Institut Laue-Langevin, 71 avenue de Martyrs, Grenoble 38042, France

^25^ Institució Catalana de Recerca i Estudis Avançats (ICREA), Passeig Lluís Companys 23, Barcelona 08010, Spain

^26^ Institute for Research in Biomedicine, The Barcelona Institute of Science and Technology, Baldiri Reixac, 10, Barcelona 08028, Spain

^27^ School of Chemical Sciences, The University of Auckland, Auckland, New Zealand

^28^ Department of Theoretical and Computational Biophysics, Max Planck Institute for Biophysical Chemistry, D-37077 Göttingen, Germany

^29^ Instituto de Química Física ‘Blas Cabrera’, Consejo Superior de Investigaciones Científicas (CSIC), Madrid, Spain

^30^ Institute of Biomembranes, Bioenergetics and Molecular Biotechnologies, National Research Council (CNR-IBIOM), Bari, Italy

^31^ University Lyon 1, ICBMS, UMR 5246 CNRS, Villeurbanne, France

^32^ Department of Chemistry, Dartmouth College, New Hampshire, USA

^33^ Department of Drug Science and Technology, Università degli Studi di Torino, Torino, Italy

^34^ Protein Data Bank in Europe, European Molecular Biology Laboratory, European Bioinformatics Institute (EMBL-EBI), Wellcome
